# Design and Use of Patient-Facing Electronic Patient-Reported Outcomes and Sensor Data Visualizations During Outpatient Chemotherapy

**DOI:** 10.2196/62711

**Published:** 2025-01-10

**Authors:** Christianna Bartel, Leeann Chen, Weiyu Huang, Qichang Li, Qingyang Li, Jennifer Fedor, Krina C Durica, Carissa A Low

**Affiliations:** 1 Department of Medicine, University of Pittsburgh, Suite 5002, 5051 Centre Avenue, Pittsburgh, PA, 15213, United States, (412) 623-5973

**Keywords:** oncology, cancer, data visualization, remote monitoring, mobile technology, patients, outpatient, chemotherapy, symptoms, side effects, cancer treatment, electronic patient-reported outcome, online, monitoring, self-management

## Abstract

This study describes patients’ interaction with a personalized web-based visualization displaying daily electronic patient-reported outcomes and wearable device data during outpatient chemotherapy.

## Introduction

Chemotherapy can cause significant symptoms that impact the quality of life [1]. Although electronic patient-reported outcome (ePRO) systems for collecting symptom ratings from patients have become increasingly common in cancer care, most of these are designed for clinicians, and fewer than half share data visualizations with the patients [2,3]. Visualization of ePRO and other data (eg, wearable device data) may help patients undergoing cancer treatment find patterns that help them to prepare for future treatment cycles, manage side effects, and have productive conversations with clinicians [4,5].

**Figure 1.**
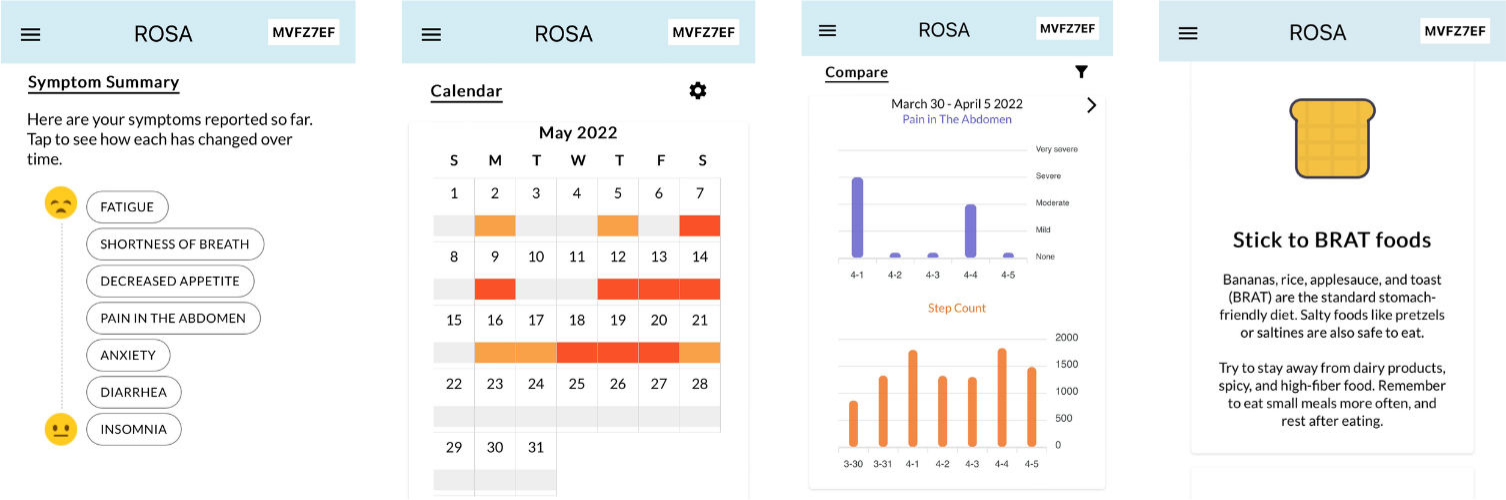
Visualizations of daily symptom ratings and wearable data with self-care resources.

As part of a prospective longitudinal National Cancer Institute–funded study to develop a remote symptom monitoring system during chemotherapy [6], we created mobile-friendly web visualizations of each patient’s daily symptom ratings and wearable device data (Figure 1). The aim of this paper is to describe patterns of use of these novel visualizations.

## Methods

### Recruitment and Study Design

Participants undergoing chemotherapy for any solid tumor and who owned smartphones were recruited from oncology clinics at a single academic center in Pittsburgh, Pennsylvania. During the study, the participants wore a Fitbit device (Inspire; Google LLC) and reported 16 symptoms commonly experienced during chemotherapy (eg, nausea, fatigue) daily, using the patient-reported version of the Common Terminology Criteria for Adverse Events [7]. The website also included evidence-based symptom self-management resources as described by Donovan et al [8].

At the time of enrollment, we provided each patient with a personalized link to their real-time visualizations; however, no instructions about viewing frequency for the visualizations or usage reminders were given during the study. At the end of the 3-month study, 141 patients completed an electronic- or paper-based 11-question survey (mean completion time was 5 min) to assess whether they used the visualizations, frequency of use, helpful information, and suggestions for improvement. Data were collected between February 2022 and April 2024.

### Ethical Considerations

The University of Pittsburgh’s Institutional Review Board approved all study activities (19070011). At the time of enrollment, all participants provided informed written consent. All data were stored in secure locations and identified only by anonymized study ID numbers. Participants received US $100 and could keep the Fitbit device (estimated value $100).

## Results

Characteristics of the participants can be found in Table 1. Survey respondents were heterogeneous in age (mean 61, SD 12; range 29-92 years), race (113/141, 80% White; 28/141, 20% other races), and cancer stage (75/135, 56% stage IV). Approximately half (76/141, 54%) of the participants accessed the link to their data visualizations. Participants with non-binary gender (n=1, 0.7%) and unknown cancer stage (n=6, 4.3%) were excluded from *χ*^2^ analysis while comparing participants who accessed their visualizations. There were no significant differences between the participants who clicked on the link and those who did not during the study in terms of mean age (*P*=.74), gender (*P*=.66), race (*P*=.50), or cancer stage (*P*=.31). Of those who accessed the platform, most (54%, 41/76) viewed it a few times (ie, less than monthly), while 13% (10/76) used it daily. The 10 daily users were within 3 months of starting chemotherapy for the first time. Most participants (58/75, 77%) found the visualizations *somewhat* or *very helpful/informative*. Few participants shared their data with family members or friends (11/141, 8%) and with others (2/141, 1%); none shared data with their providers or other patients. Participant–suggested improvements included reminders to view graphs and the ability to enter treatment and surgery dates.

**Table 1.** Participant characteristics.

Characteristics	Overall (N=141)	Did not click the link (n=65)	Clicked the link (n=76)	Test statistic (*df*)^a^	*P* value
Age (years), mean (SD)	61 (12)	61 (13)	60 (11)	*t* (130.1)=0.68	.74
Gender, n (%)				*χ*^*2*^(1)=0.2	.66^b^
Female	94 (66.7)	45 (69.2)	49 (64.5)		
Male	45 (31.9)	19 (29.2)	26 (34.2)		
Non-binary	1 (0.7)	0 (0)	1 (1.3)		
Unknown	1 (0.7)	1 (1.5)	0 (0)		
Race, n (%)				*χ*^*2*^(1)=0.45	.5
White	113 (80.1)	50 (76.9)	63 (82.9)		
Others^c^	28 (19.8)	15 (23.1)	13 (17.1)		
Stage IV cancer, n (%)				*χ^2^*(1)=1.04	.31^d^
Yes	75 (53.2)	39 (60.0)	36 (47.4)		
No	60 (42.5)	25 (38.5)	35 (46.0)		
Unknown	6 (4.3)	1 (1.5)	5 (6.6)		

a Welch two sample *t*-test and Pearson’s *χ*2 test were used, as appropriate; degrees of freedom (*df*) are provided in parentheses.

b Participants with non-binary or unknown gender were excluded from this test.

c Other race category included Black or African-American (n=24), Asian (n=2), and more than one race (n=2).

d Participants with unknown cancer stage were excluded from this test.

## Discussion

### Overview

Providing real-time visualizations of ePRO and activity data throughout chemotherapy may help patients anticipate and manage symptoms effectively and potentially identify patterns between activity or other sensor data and symptoms. These preliminary findings suggest that patients are motivated to view their data, and these visualizations were accessible to patients of different ages, races, and cancer stages. Daily users, who were mostly new to chemotherapy, may have higher levels of anxiety and a greater need for health information [9]. Future studies should investigate the potential benefits of patient-facing visualizations for patients beginning chemotherapy.

### Limitations

The visualizations and other website content were developed as part of an ancillary project in an ongoing study, and participants received no instructions or reminders regarding website usage. Survey respondents represented a subset (141/158, 89%) of participants, who received personalized visualizations, and the results may be influenced by selection, response, and recall biases.

### Conclusion

This study describes initial efforts to share real-time ePRO and wearable device data visualizations with patients undergoing chemotherapy. Further research is needed to improve the usability of data visualizations and evaluate their impact on symptom management, self-efficacy, and other outcomes.

## Abbreviations

ePRO: electronic patient-reported outcomes
